# *De novo* transcriptome sequencing and digital gene expression analysis predict biosynthetic pathway of rhynchophylline and isorhynchophylline from *Uncaria rhynchophylla*, a non-model plant with potent anti-alzheimer’s properties

**DOI:** 10.1186/1471-2164-15-676

**Published:** 2014-08-12

**Authors:** Qianqian Guo, Xiaojun Ma, Shugen Wei, Deyou Qiu, Iain W Wilson, Peng Wu, Qi Tang, Lijun Liu, Shoukun Dong, Wei Zu

**Affiliations:** College of Agriculture, Northeast Agricultural University, Harbin, 150030 China; College of Life Science, Agriculture and Forest, Qiqihar University, Qiqihar, 161006 China; Institute of Medicinal Plant Development, Chinese Academy of Medical Sciences, Beijing, 100193 China; Yunnan Branch Institute of Medicinal Plant Development, Chinese Academy of Medical Sciences, Jinghong, 530023 China; Guangxi Medicinal Botanical Garden, Nanning, 530023 China; State Key Laboratory of Tree Genetics and Breeding, The Research Institute of Forestry, Chinese Academy of Forestry, Beijing, 100091 China; CSIRO Plant Industry, PO Box 1600, Canberra, ACT 2001 Australia; Hunan Agricultural University, Changsha, 410128 China

**Keywords:** Transcriptome, DGE, *Uncaria rhynchophylla*, Rhynchophylline, Isorhynchophylline, Terpene indole alkaloid biosynthesis

## Abstract

**Background:**

The major medicinal alkaloids isolated from *Uncaria rhynchophylla* (gouteng in chinese) capsules are rhynchophylline (RIN) and isorhynchophylline (IRN). Extracts containing these terpene indole alkaloids (TIAs) can inhibit the formation and destabilize preformed fibrils of amyloid β protein (a pathological marker of Alzheimer’s disease), and have been shown to improve the cognitive function of mice with Alzheimer-like symptoms. The biosynthetic pathways of RIN and IRN are largely unknown.

**Results:**

In this study, RNA-sequencing of pooled *Uncaria* capsules RNA samples taken at three developmental stages that accumulate different amount of RIN and IRN was performed. More than 50 million high-quality reads from a cDNA library were generated and *de novo* assembled. Sequences for all of the known enzymes involved in TIAs synthesis were identified. Additionally, 193 cytochrome P450 (CYP450), 280 methyltransferase and 144 isomerase genes were identified, that are potential candidates for enzymes involved in RIN and IRN synthesis. Digital gene expression profile (DGE) analysis was performed on the three capsule developmental stages, and based on genes possessing expression profiles consistent with RIN and IRN levels; four CYP450s, three methyltransferases and three isomerases were identified as the candidates most likely to be involved in the later steps of RIN and IRN biosynthesis.

**Conclusion:**

A combination of *de novo* transcriptome assembly and DGE analysis was shown to be a powerful method for identifying genes encoding enzymes potentially involved in the biosynthesis of important secondary metabolites in a non-model plant. The transcriptome data from this study provides an important resource for understanding the formation of major bioactive constituents in the capsule extract from *Uncaria*, and provides information that may aid in metabolic engineering to increase yields of these important alkaloids.

**Electronic supplementary material:**

The online version of this article (doi:10.1186/1471-2164-15-676) contains supplementary material, which is available to authorized users.

## Background

Alzheimer is a common disease that threatens the health of the elderly [1–3]. *Uncaria* a member of the Rubiaceae family, has long been used in traditional Chinese medicine to treat hypertension, convulsions, tremor, and stroke [4]. Indole alkaloids present in aqueous solution have been shown to interfere with fiber formation of β-amyloid protein (Aβ) and destabilize preformed Aβ fibrils, a pathological marker of Alzheimer’s disease [5]. An ethanol extract of *Uncaria* was found to improve the cognitive impairment of Alzheimer’s disease caused by D-galactose in mice [6]. RIN and IRN two major alkaloid chemicals that are synthesized in *Uncaria* capsules, can inhibit the activation of microglia and reduce Aβ-induced death in cell lines used to study neuronal differentiation (PC12) [7]. Also RIN reduces Aβ cytotoxicity by inhibiting intracellular calcium overloading and tau protein hyperphosphorylation [8, 9]. Extracts from *Uncaria* have the potential for delaying or alleviating the symptoms of Alzheimer’s diseases that could significantly improve the quality of life of patients.

RIN and IRN belong to TIAs. TIAs are a diverse class of natural products that comprise over 2000 members, many of which possess significant physiological activity. Most TIAs are found in three dicotyledon plant families: Apocynaceae, Rubiaceae and Loganiaceae, and are thought to be part of a plants chemical defense against pests [10]. TIAs have a wide variety of different molecular structures and biological activities [11]. Notable TIAs include vincristine and vinblastine of *Catharanthus roseus* that have anti-tumor activity. Vinblastine is clinically used for the treatment of leukemia, Hodgkin’s lymphoma and other cancers [12–15]. Ajmaline of *Rauwolfia serpentina* can block the sodium channel and resist arrhythmia [15–17]. Whereas, Yohimbine is antagonist of α-2-adrenoceptor and a potential drug for the treatment of erectile dysfunction [18, 19]. Camptothecin of *Ophiorrhiza pumila* and *Camptotheca acuminata* is a topoisomerase enzyme inhibitor, and has anti-cancer effect [20, 21]. Quinine of *Cinchona* species is a known anti-malarial drug [22, 23]. TIAs production in plants is generally low and difficult to synthesize chemically, limiting the utility of these therapeutic chemicals unless production is increased by metabolic engineering [11]. However, metabolic engineering requires knowledge of the TIAs biosynthesis pathway which is only well understood for a few, such as vindoline and ajmaline.

**Figure 1:**
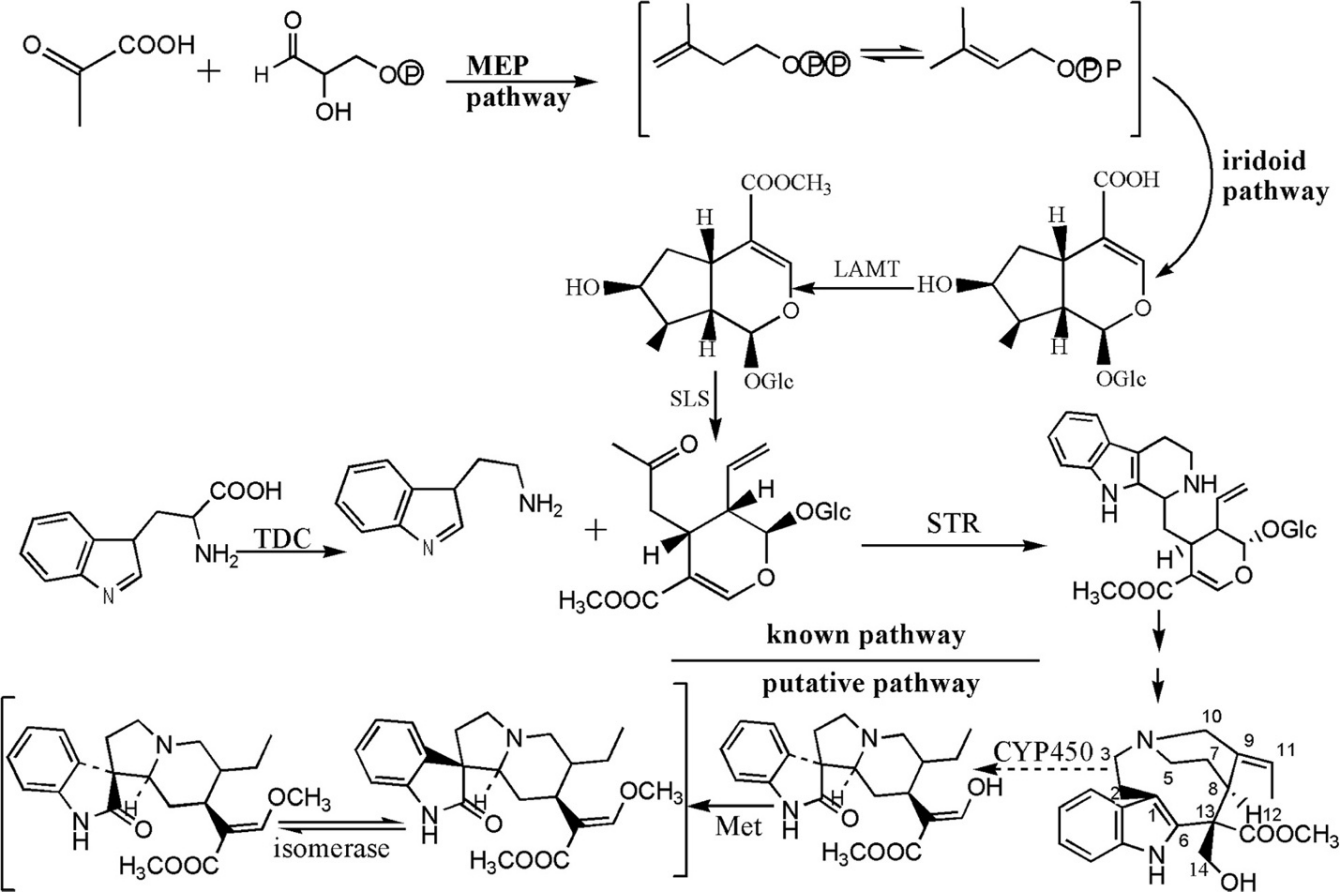
**Putative rhynchophylline and isorhynchophylline biosynthesis pathway in** ***Uncaria rhynchophylla.*** MEP pathway: “non-mevalonate” pathway; TDC: tryptophan decarboxylase, EC:4.1.1.28; STR: strictosidine synthase, EC:4.3.3.2; P450: cytochrome P450, EC:1.14.-.-.

TIAs biosynthesis involve multiple and complex metabolic pathways. All TIAs are derived from tryptophan and secologanin. Tryptophan decarboxylase (TDC; EC 4.1.1.28), a pyridoxal dependent enzyme converts tryptophan to tryptamine. Isopentenyl diphosphate (IPP) the precursor for all terpenoids is produced by the triose phosphate/pyruvate or “non-mevalonate” pathway (MEP/DOXP pathway) [24, 25]. In the first committed step of iridoid terpene biosynthesis, geraniol, derived from IPP, is hydroxylated by geraniol-10-hydroxylase (G10H; EC 2.5.1.1) [26, 27]. Oxidation of the iridotrial aldehyde to the carboxylic acid is followed by esterification and glucosylationto, yield deoxyloganin; subsequent hydroxylation of deoxyloganin yields loganin. Secologanin is then generated by oxidative cleavage of loganin by the enzyme secologanin synthase (SLS; EC 1.3.3.9) [28–33]. Biosynthesis of secologanin is thought to be potential rate-limiting step in TIAs biosynthesis. Tryptamine of tryptophan metabolic pathway via strictosidine synthase (STR; EC 4.3.3.2) catalysis forms strictosidine [34], and then enters TIAs biosynthetic pathway [35]. This part of the synthesis processes is the best characterized, and the genes encoding the enzymes are known. However each individual TIAs has a synthetic branch, which is started at strictosidine. Through four variable precursors, (4,21-Dehy drogeissoschizine, cathenamine, 19-epi-cathenamine and stemmadenine), each active substance enters the branch path. From structural analysis of RIN and IRN, they may be transformed from Stemmadenine (Figure 1). This speculation is according to the up-stream pathway of TIAs synthesis, which is the biosynthetic process of secologanin [36, 37]. Stemmadenine synthesized RIN and IRN through the chemical reactions of oxidation and methyl transfer. This process requires three kinds of enzymes; oxidoreductases, methyltransferases and isomerases. The genes coding for oxidoreductases, methyltransferases and isomerases are defined as “late step genes” of RIN and IRN biosynthesis.

Next generation sequencing technologies have proved to be rapid and cost-effective means to analyze the genome and transcriptome in non-model species. In this study, knowledge of the rapid accumulation periods of RIN and IRN in *Uncaria* was used to find potential candidates in the synthesis of these TIAs. Using transcriptome sequencing, *de novo* assembly and DGE analysis, candidate genes encoding putative enzymes responsible for late steps in RIN and IRN synthesis were identified. Our results lay a foundation for construction of the RIN and IRN biosynthetic pathways that will in turn aid the study of the regulation and metabolic engineering of RIN and IRN biosynthesis.

## Results

### *De novo*transcriptome sequencing of capsule

**Figure 2:**
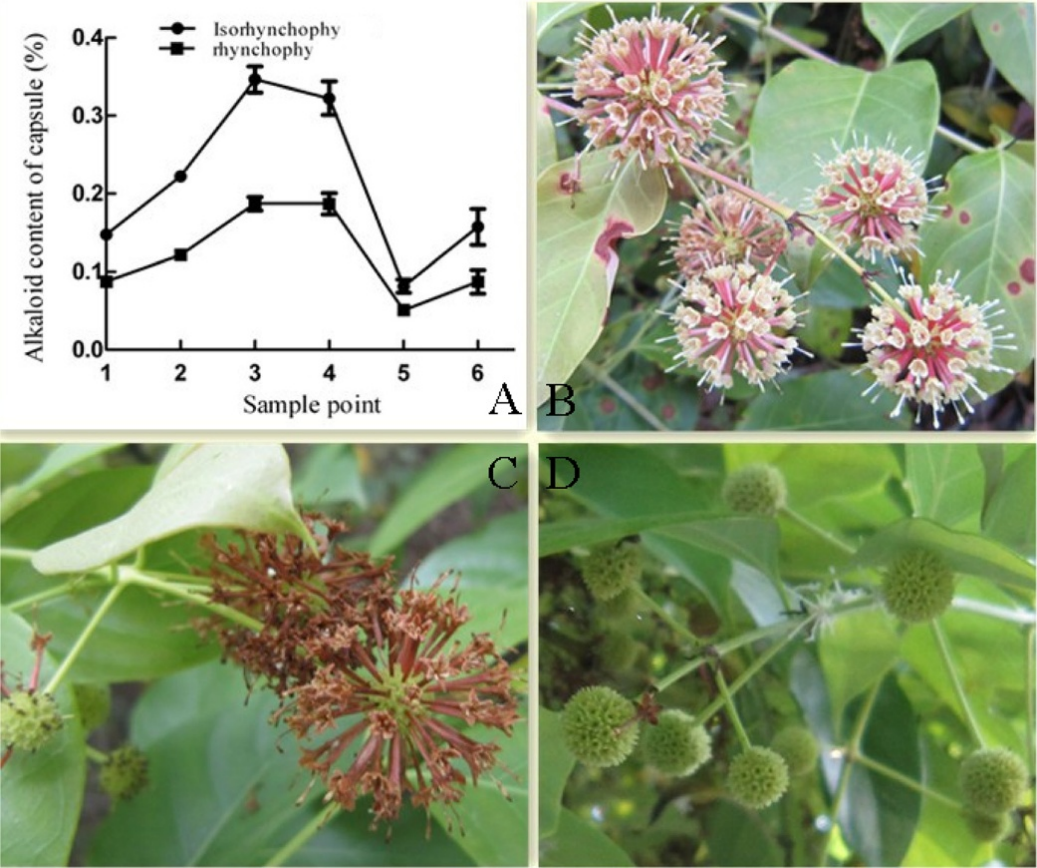
**The RIN and IRN content changes of** ***Uncaria*** **capsule from the fallen petal to yellow maturity (A) and biomorph of the sample point 1,3,5 (B, C, D).**

We compared RIN and IRN content throughout the development of *Uncaria’s* capsule, from fallen petal (designated capsule 1, sample point #1) to maturity (designated capsule 3, sample point #5) (Figure 2). RIN and IRN content changed dramatically during the development of the *Uncaria* capsule. The two TIAs accumulate in the capsules were detected in capsule 1 (fallen petal), until a maximum is reached at sample point 3 (designated capsule 2) 20 days later, and then drops to its lowest levels at capsule maturity (designated capsule 3) after another 20 days (Figure 2). To obtain an overview of the *Uncaria* capsule transcriptome in the process of RIN and IRN accumulation, a cDNA library was generated from an equal mixture of RNA isolated from capsule 1 (starting point for RIN and IRN accumulation), capsule 2 (peak RIN and IRN content), and capsule 3 (RIN and IRN content is at its lowest). The library was sequenced using the Illumina HiSeq™ 2000 platform. 51 million raw reads were obtained which had 50,159,521 clean reads (97.49% of all raw reads) (in Additional file 1: Table S1), which were used for all subsequent analysis.

With the increase of the sequencing length, sequencing error rates will rise [38, 39], but the error rate does not exceed 1% (in Additional file 2: Figure S1). 6 bp random primers were used in reverse transcription. The incomplete binding of random primers and template could cause sequencing errors of the first six nucleotides position [39, 40] (in Additional file 2: Figure S1, S2). Clean reads were spliced into transcripts by trinity software (v2012-10-05) [41], and 100,940 transcripts were obtained. Transcript length was an index which measured with splicing effect. To a certain extent, N50 can evaluate splicing integrity [42]. The average length of transcript was 1,181 bp, the longest transcript was 14,101 bp, the shortest transcript was 201 bp (N50 was 1970 bp, N90 was 503 bp). From these transcripts, 55,523 unigenes were identified, with an average length of 717 bp, the longest unigene was 13,307 bp, the shortest unigene 201 bp (N50 was 1242 bp, N90 was 280 bp). The length statistics of spliced transcripts and unigenes were shown in Additional file 1: Table S2.

### Annotation of gene function and CDS prediction

In order to assign accurate annotation information to unigenes, multiple databases were interrogated including; the NCBI database Non-redundant protein sequences (Nr), the manually annotated and curated protein sequence database (Swiss Prot), NCBI nucleotide sequences (Nt), Protein Family (Pfam), Clusters of Orthologous Groups of proteins (COG), Gene Ontology (GO) and Kyoto Encyclopedia of Genes and Genomes Ortholog database (KO). Annotation results of unigenes are shown in Table 1.

**Table 1:** **Annotation result statistics between unigenes and databases**

Databases	Number of Unigene	Percent (%)
Nr	28,715	51.72
Swiss-Prot	19,984	35.99
Nt	10,863	19.56
Pfam	21,083	37.97
COG	11,150	20.08
GO	26,692	48.07
KO	12,256	22.07

**Figure 3:**
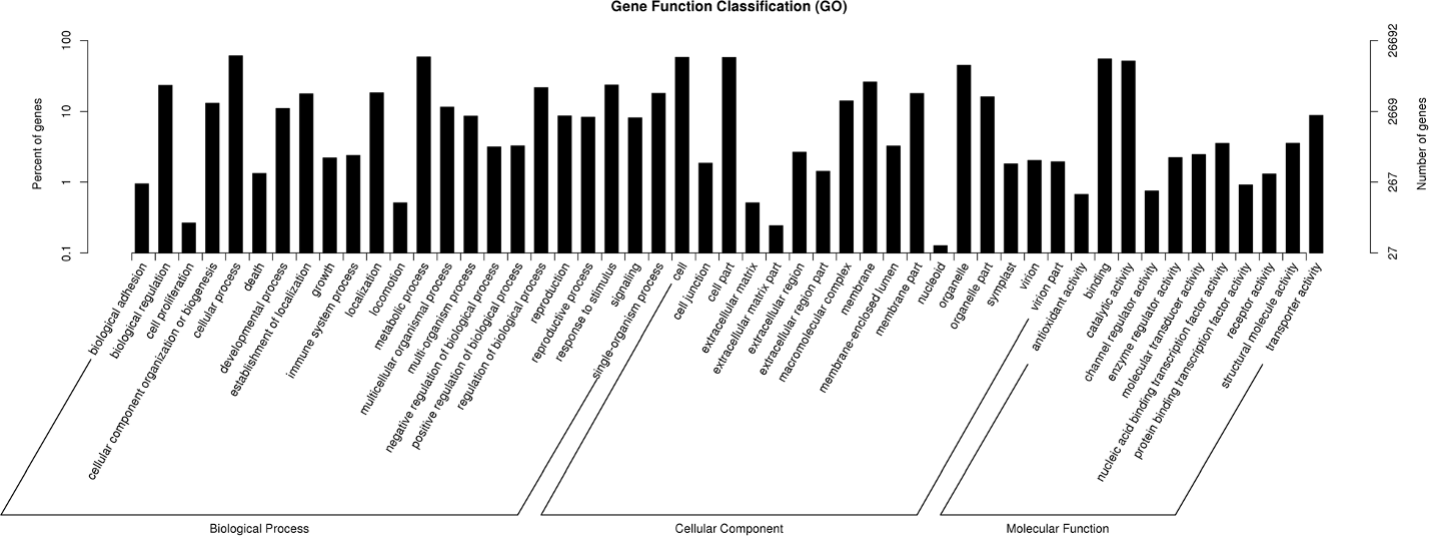
**Histogram of Gene Ontology classification.** The results are summarized in three main categories: biological process, cellular component and molecular function. The right y-axis indicates the number of genes in a category. The left y-axis indicates the percentage of a specific category of genes in that main category.

**Figure 4:**
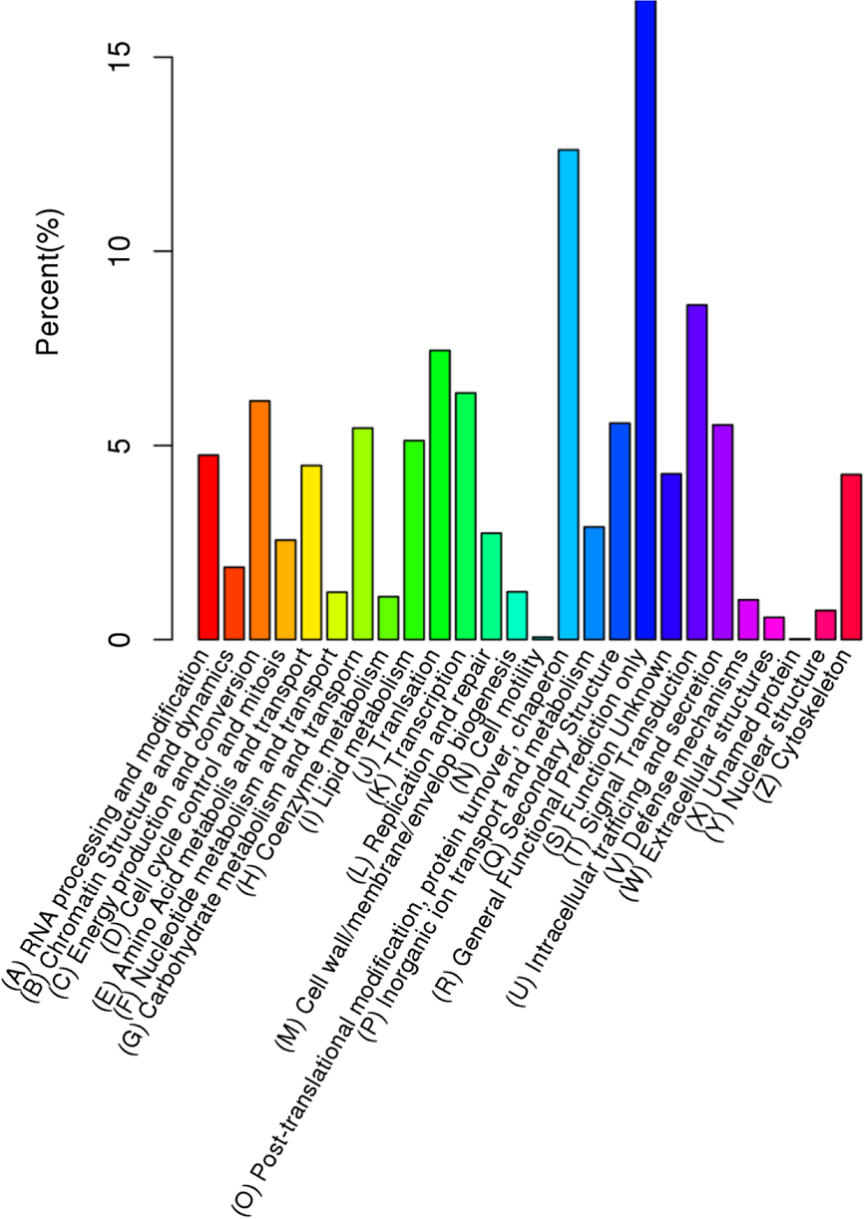
**Histogram of unigene KOG classification.** The x-axis indicates 26 groups of KOG. The y-axis indicates the percentage of the number of genes annotation under the group in the total number of genes annotation.

**Figure 5:**
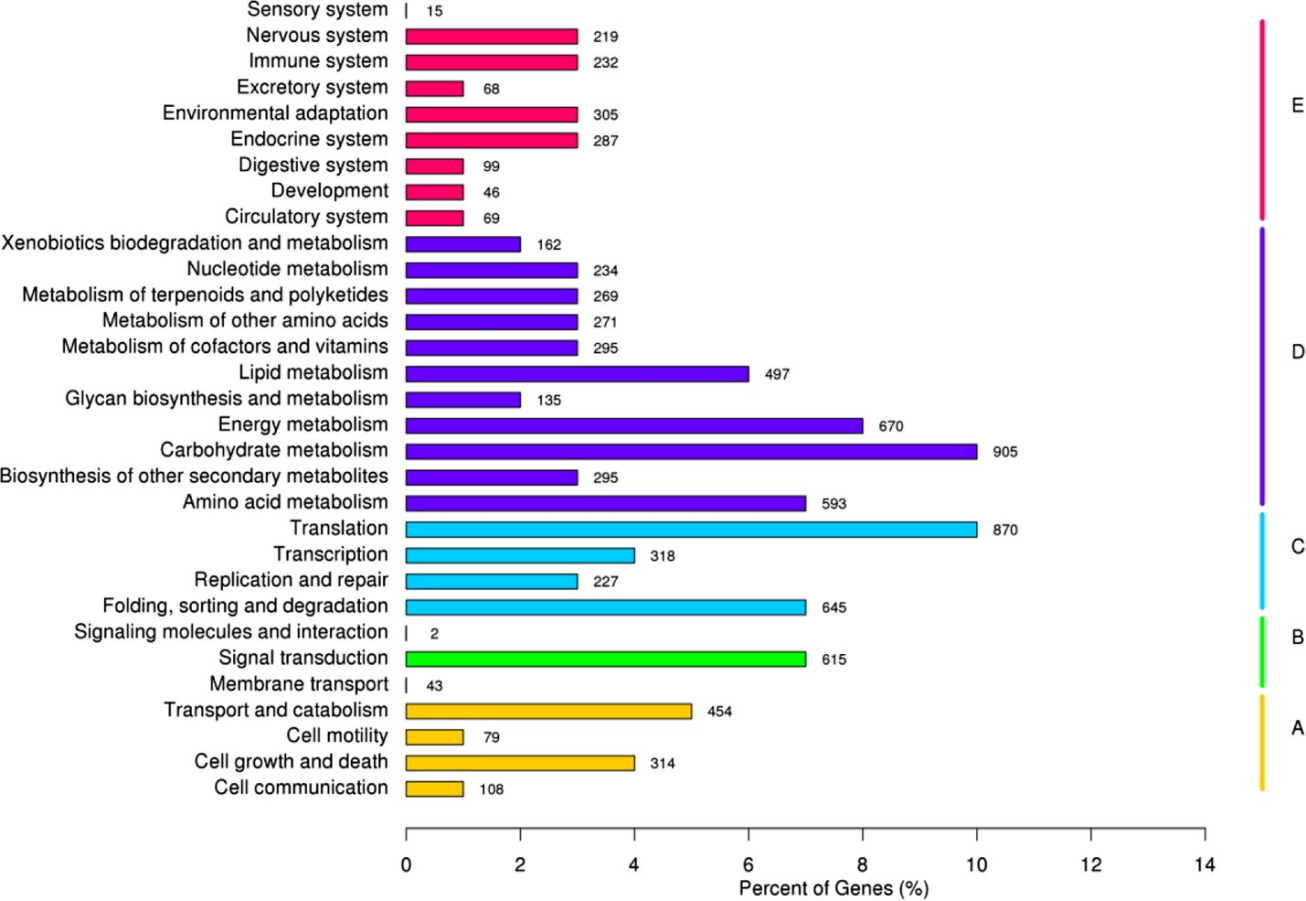
**Functional classification and pathway assignment of unigenes by KEGG.** The results are summarized in five main categories: **A**, Cellular Processes; **B**, Environmental Information Processing; **C**, Genetic Information Processing; **D**, Metabolism; **E**, Organismal Systems. The y-axis indicates the name of the KEGG metabolic pathways. The x-axis indicates the percentage of the number of genes annotation under the pathway in the total number of genes annotation.

GO is a classification system to describe the properties of the organism genes and their products, including Biological Process, Cellular Component and Molecular Function [43]. Through alignment of GO databases, 26,692 unigenes were annotated to 57 terms of GO classification (Figure 3). Among Biological Process, ‘immune system process [GO:0002376]’ was annotated with 637 unigenes, ‘response to stimulus [GO:0050896]’ was annotated with 6328 unigenes, ‘metabolic process [GO:0008152]’ was annotated with 15636 unigenes.KOG is a classification system based on orthologous genes. Orthologous genes have the same function and common ancestor. 11,150 unigenes were annotated to 26 groups by KOG database (Figure 4). (R) General Functional Prediction Only annotated 1869 unigenes at most, and (X) Unnamed protein annotated 2 unigenes at least. (G) Carbohydrate metabolism and transport was annotated with 607 unigenes, (V) Defense mechanisms was annotated with 113 unigenes (Figure 4).KO is a network diagram of cell metabolic pathways, including metabolism, genetic information processing, environmental information processing, cellular processes, organismal systems and human diseases. In this study, 12,256 unigenes were annotated by alignment with the KO database. Among them; 4,326 unigenes were annotated to Metabolism (35.30% of the total), 660 to Environmental Information Processing (5.39%), 2,060 to Genetic Information Processing (16.80%), 1,340 to Organismal Systems (10.93%), 955 to Cellular Processes (7.79%). According to the KO annotation, removing unigenes associated with the Human Diseases, the remaining 8,745 unigenes (15.75% of all unigenes) were classified to 31 KEGG pathways (Figure 5). Among them; 269 were annotated to Metabolism of terpenoids and polyketides pathway, 74 unigenes involved in Terpenoid backbone biosynthesis [PATHWAY: ko00900], 17 unigenes involved in Monoterpenoid biosynthesis [PATHWAY: ko00902], 593 unigenes were annotated to Amino acid metabolism pathway, 54 unigenes involved in Phenylalanine, tyrosine and tryptophan biosynthesis [PATHWAY: ko00400], 295 unigenes were annotated to Biosynthesis of other secondary metabolites pathway, 8 unigenes involved in Indole alkaloid biosynthesis [PATHWAY: ko00901].

Open Reading Frame (ORF) was obtained by comparing annotation results of unigenes with the different databases, the unigenes were then determined using Coding Sequence (CDS) according to the standard codon table [38]. If the results from different databases were contradictory, priority was given to Nr, Swiss-prot and KEGG GENES respectively. The unigenes that failed to match the database information the predicted protein CDS was determined using estscan software [44]. The CDS of 28,611 unigenes (51.52% of all unigenes) could be predicted using the annotation databases whereas 16,566 unigenes (29.84% of all unigenes) CDS predictions were made using estscan software.

### Genes involved in the biosynthesis of TIAs

TIAs biosynthesis involves three upstream metabolic pathways, including MEP pathway, iridoid glycosides pathway and shikimate pathway [45]. Secologanin is a natural active substance and iridoid glycoside product of TIAs biosynthetic precursor, but its synthesis process is not yet understood. Another precursor tryptamine; is generated by tryptophan using the shikimate pathway by catalysis with TDC. TDC is communication node between primary metabolism and secondary metabolism. Unigenes annotated by comparison with the Nr database comparison that could encode enzymes related the above pathways are listed in Additional file 1: Table S3. All putative oxidoreductases that could represent genes involved in the late steps in RIN and IRN synthesis are listed in Additional file 1: Table S4 (A-C).

The MEP pathway is branch of Terpenoid backbone biosynthesis [PATHWAY: ko00900] and the iridoid glycosides pathway is branch of Monoterpenoid biosynthesis [PATHWAY: ko00902], The product of phenylalanine, tyrosine and tryptophan biosynthesis [PATHWAY: ko00400] enters indole alkaloid biosynthesis [PATHWAY: ko00901] via TDC and STR. Putative unigenes associated with TIAs synthesis were annotated by KO database (in Additional file 1: Table S5). The *Uncaria* capsule transcriptome data contained all genes encoding every enzyme of the MEP pathway and shikimate pathway. However, there are deficiencies associated with the databases as the Monoterpenoid biosynthesis [PATHWAY: ko00902] in KO is only aligned to myrcene/ocimene synthase (EC 4.2.3.15). In the KEGG pathway, TDC belongs to indole alkaloid biosynthesis [PATHWAY: ko00901], which is only aligned to STR, not aligned to the TDC. The reason may be few studies were conducted with TIAs biosynthesis, and reference data was insufficient.

### Capsule DGE analysis at different periods

**Figure 6:**
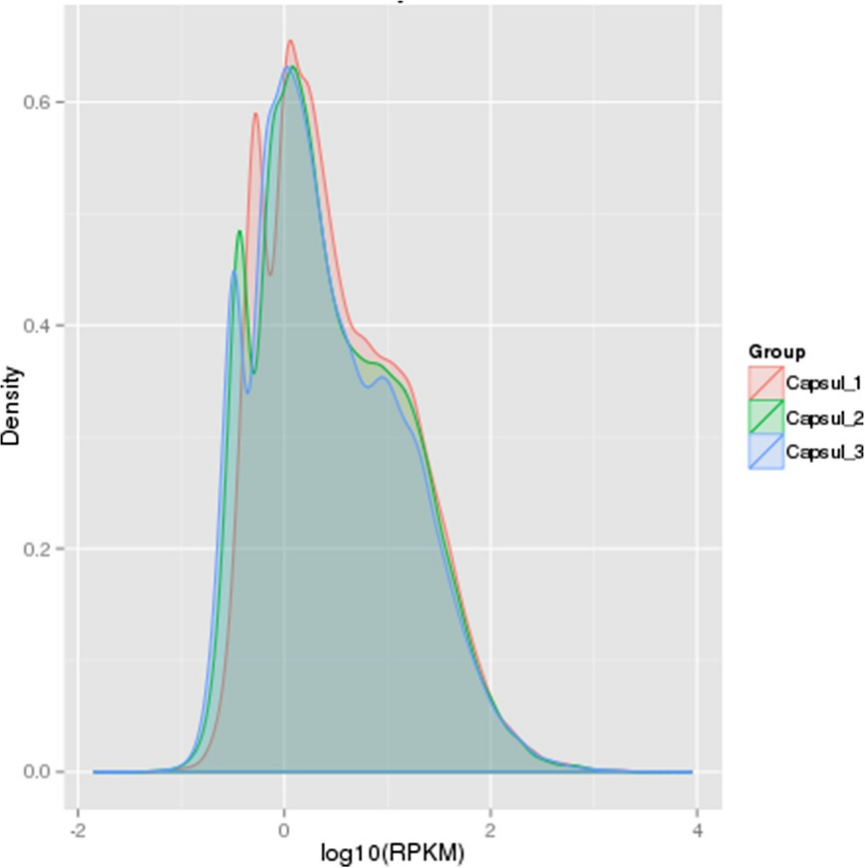
**Capsule RPKM density distribution at different periods.** The y-axis indicates the density values of log_10_ (RPKM). The x-axis indicates the log_10_(RPKM) values of genes. Red, green and blue represent Capsule 1, Capsule 2 and Capsule 3 respectively.

DGE sequencing analysis of capsule 1, capsule 2 and capsule 3 was performed by Illumina HiSeq™ 2000 sequencing platforms, respectively. Capsule 1 produced 9,270,290 clean reads, capsule 2 produced 13,298,879 clean reads, and capsule 3 produced 15,331,847 clean reads. The clean reads of the samples were mapped to the reference sequence derived from our *Uncaria* transcriptome data, and obtained from the annotation information of each sample using RSEM software [46]. Total mapped reads were capsule 1 8,529,453 (92%), capsule 2 12,258,147 (92%), and capsule 3 14,047,840 (92%) with approximately 47,000 unigenes annotated in each sample. To estimate the level of gene expression the reads count were transformed into Reads Per Kilo bases per Million mapped Reads (RPKM) [47]. RPKM is the number of reads per million reads from one gene per thousand bases, considering the effect of sequence length and depth of RNA-seq process on the reads count. The size of RPKM value therefore reflects the abundance of the gene expression and we defined RPKM > 0.3 as the threshold of significant gene expression. The density distribution of RPKM can test gene expression profiles of samples; comparison chart of gene expression profiles among three samples was showed in Figure 6. Among them, the common genes of three samples reached 38,592, characteristic genes of capsule 1 were 1,687, characteristic genes of capsule 2 were 1,901 and characteristic genes of capsule 3 were 1,986.

### Analysis of differentially expressed genes

**Figure 7:**
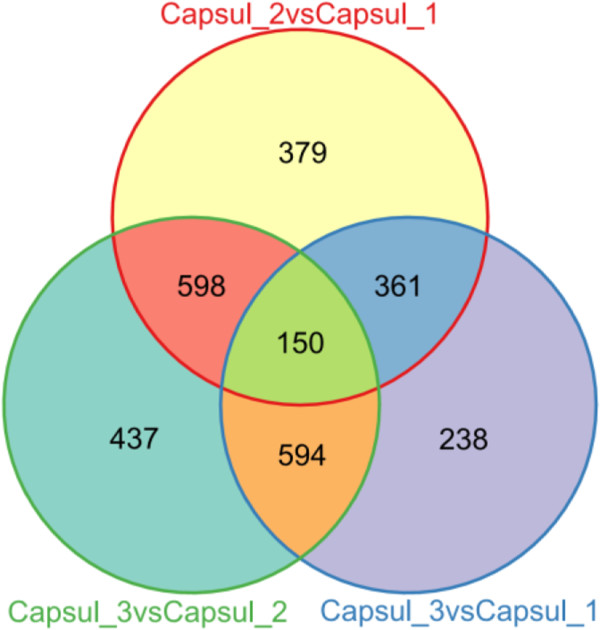
**Venn diagram of differentially expressed genes.** The sum of the numbers in each large circle represents total number of differentially expressed genes between combinations, the overlap part of the circles represents common differentially expressed genes between combinations.

Read count data was used to analyze the difference of gene expression. Since only one biological samples was sequenced, in order to avoid possible high false positive rates we first used trimmed mean of M values (TMM) to standardize data processing [48], then took advantage of DEGseq to analyze differentially expressed genes; the analysis conditions were qvalue <0.005 & log2 (foldchange) >1 [49–51]. There were 1,488 differentially expressed genes identified between capsule 2 and capsule 1, with 803 genes were up-regulated and 685 genes were down-regulated; 1,343 differentially expressed genes between capsule 3 and capsule 1, 749 genes were up-regulated and 549 genes were down-regulated; 1,779 differentially expressed genes in capsule 3 vs capsule 2, with 865 genes were up-regulated and 914 genes were down-regulated. Comparison between differentially expressed genes between the three samples is shown in Figure 7. Capsule 3 vs capsule 2 comparisons had the most specific differentially expressed genes. Interestingly, capsule 3 vs capsule 2 and capsule 2 vs capsule 1 had 748 common differentially expressed genes, capsule 3 vs capsule 2 and capsule 3 vs capsule 1 had 744 common differentially expressed genes, the two were very close.

Differentially expressed genes of from the DGE analysis were further analyzed using GO and KEGG enrichment in order to determine their potential function and within a metabolic pathway. GO enrichment classification using GOseq [52]. GO classification of differentially expressed genes between the different capsule stages is showed in Additional file 2: Figure S3 (A-C). 419 differentially expressed genes of capsule 2 vs capsule 1 were classified into the biological process, and 133 genes were identified as involved in oxidation-reduction process [GO: 0055114]. 703 genes were classified into cellular component. 903 genes were classified into molecular function, 903 genes involved in catalytic activity [GO:0003824], 137 genes involved in oxidoreductase activity [GO: 0016491]. 588 genes among differentially expressed genes of capsule 3 vs capsule 1 were classified into the biological process, 226 genes involved in oxidation-reduction process [GO: 0055114]. 507 genes were classified into cellular component, 765 genes were classified into molecular function, 240 genes participated in oxidoreductase activity [GO: 0016491]. 657 genes among differentially expressed genes of capsule 3 vs capsule 2 were classified into the biological process, 255 genes involved in oxidation-reduction process [GO: 0055114]. 1301 genes were classified into cellular component, 841 genes were classified into molecular function, 272 genes participated in oxidoreductase activity [GO: 0016491].

KEGG enrichment classification (hypergeometric test) was used to identify significant enrichment pathways of differentially expressed genes between the different stages of *Uncaria* capsule development using a False Discovery Rate (FDR) ≤0.05. Flavonoid biosynthesis [PATHWAY: ko00941], phenylpropanoid biosynthesis [PATHWAY: ko00940], phenylalanine metabolism [PATHWAY: ko00360] and biosynthesis of secondary metabolites [PATHWAY: ko01110] were significantly enriched pathways among the differentially expressed genes between the capsule stages. Indole alkaloid biosynthesis [PATHWAY:ko00901] was enriched in capsule 3 vs capsule 2, but did not reach significant level (FDR ≤0.05) [in Additional file 2: Figure S4 (A-C)].

### Analysis of genes with homology to known genes involved in TIA biosynthesis

There were 68 unigenes that may be involved in the TIA biosynthesis due to their homology to genes known to be involved in TIA from other species by NCBI Nr comparisons [53]. To narrow down this list we looked for genes that showed expression profiles consistent with changes in RIN and IRN content during capsule development. As previously mentioned RIN and IRN content is strongly elevated between capsule 1 and capsule 2, then decreased significantly between capsule 2 and capsule 3. RIN and IRN content is also lower in capsule 3 than that present in capsule 1. 17 of the putative TIAs biosynthesis genes showed significantly up or down-regulated expression (in Additional file 1: Table S6). However, only ten genes had expression profiles either similar or inverse to RIN and IRN accumulation in at least 2 stages. Anthranilate synthase (AS; EC 4.1.3.27) (comp12708_c0) and loganin acid methyltransferase (LAMT; EC 2.1.1.50) (comp33316_c0) expression profiles matched of the direction of RIN and IRN content change, whereas SLS (comp35216_c0) had the inverse expression profiles. The expression profiles of TDC (comp34949_c0), G10H (comp33830_c0) and STR (comp16908_c0 and comp38465_c1) had two phases consistent with RIN and IRN changes. A total of 64 unigenes of KO annotation involved in the synthesis of TIAs were based on enzymes of KEGG pathway. DGE analysis found that 11 unigenes showed significantly up/down-regulated expression changes (in Additional file 1: Table S7). AS (comp12708_c0) expression changes with direction of RIN and IRN content change was consistent. The up/down regulated expression changes of 3-dehydroquinate dehydratase/shikimate dehydrogenase (EC 4.2.1.10) (comp22817_c0) and STR (comp16908_c0) with direction of RIN and IRN content change, two phases were consistent.

### Analysis of putative genes involved in the late steps of RIN and IRN biosynthesis

Enzymes classes putatively involved of biosynthesis of RIN and IRN include oxidoreductase, transferase, and isomerase. CYP450 is one of the oldest protein families, has catalytic oxidation function of carbon-carbon bond, alkyl hydroxylation and hydroxyl oxidation, and plays an important role in plant secondary metabolites synthesis process [54–57]. Oxidoreductases involved in RIN and IRN synthesis are therefore likely to come from the CYP450 family. There are 30 varieties of methyltransferase but only o-methyl transferase is likely to be involved in RIN and IRN [58, 59]. Isomerase are relatively unknown, however isomers conversion between RIN and IRN could be realized through enzyme catalysis, as natural transformation of the chemical equilibrium is likely to be small.

From the *Uncaria* transcriptome data, a total of 193 CYP450, 280 methyltransferase and 144 isomerase unigenes were identified. DGE analysis showed 29 CYP450s were significantly up/down-regulated during capsule development (in Additional file 1: Table S8A). Only the expression of 1 unigene (comp28593_c0) matched changes in RIN and IRN content. The expression profiles of 9 unigenes (comp21921_c0, comp26673_c0, comp30485_c0, comp31075_c0, comp32966_c0, comp34791_c1, comp35950_c0, comp37580_c0 and comp38378_c0) had similar to RIN and IRN content in two stages. 21 methyltransferase unigenes possessed significantly differential expression in the process of capsule development (in Additional file 1: Table S8 B). 1 unigene (comp33316_c0) expression profile was consistent to RIN and IRN content. Whereas, the expression profile of 6 unigenes (comp12388_c0, comp29880_c0, comp34948_c0, comp36199_c0, comp36942_c0, and comp41847_c0) where similar to RIN and IRN content in two stages. 12 unigenes putatively encoding isomerases had significantly altered expression in the process of capsule RIN and IRN content change (in Additional file 1: Table S8C), with 3 unigenes (comp22426_c0, comp28178_c0 and comp38594_c0) showing changes consistent with the TIAs RIN and IRN content in two stages.

**Figure 8:**
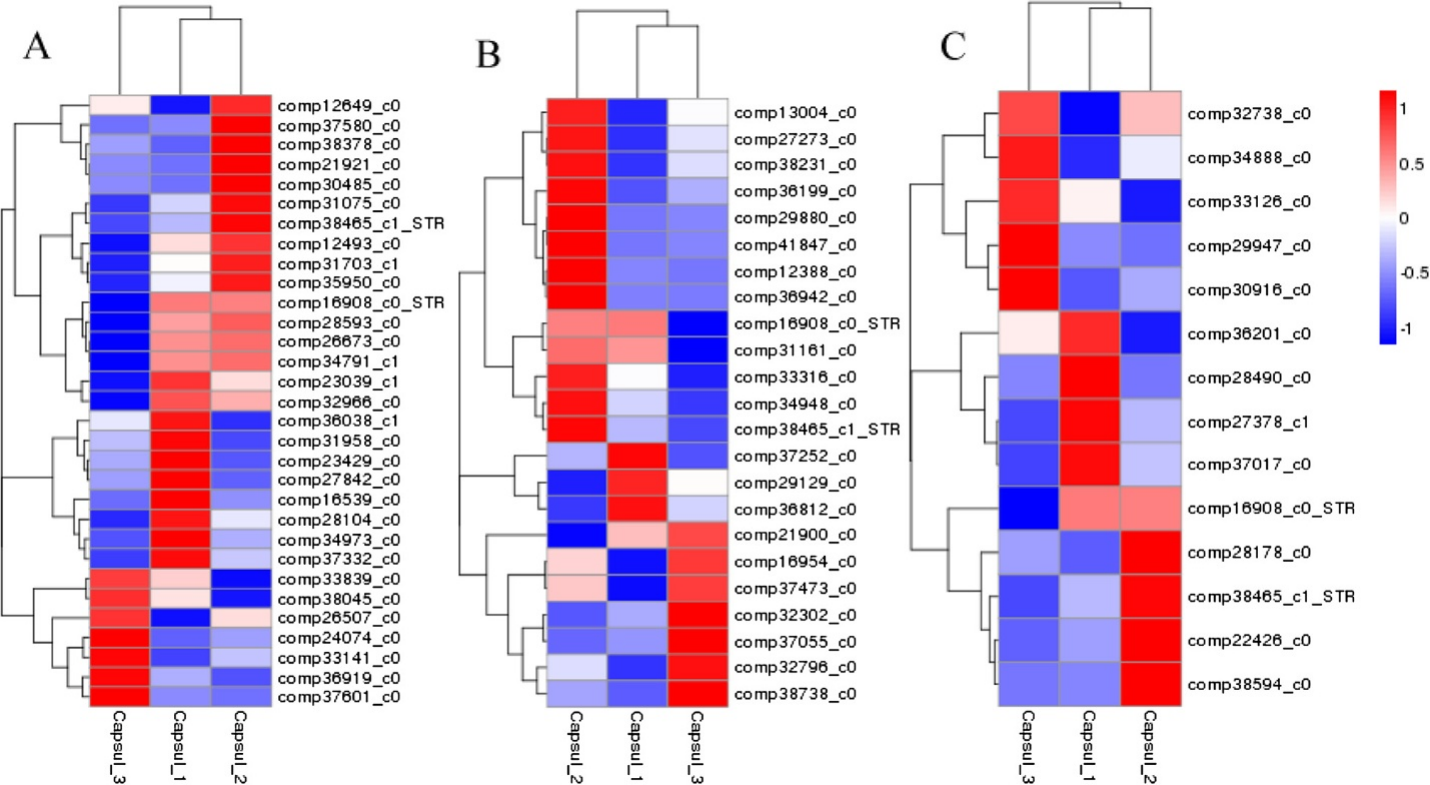
**Expression profiles clustering of CYP450, methyltransferase and isomerase genes from A to C respectively at three different capsule developmental stages.** Hierarchical clustering of expression data for 29 candidate CYP450 genes, 21 candidate methyltransferase genes and 12 candidate isomerase genes respectively using STR_Nr_ as reference profiles. Expression ratios are expressed as Log 10 values.

**Figure 9:**
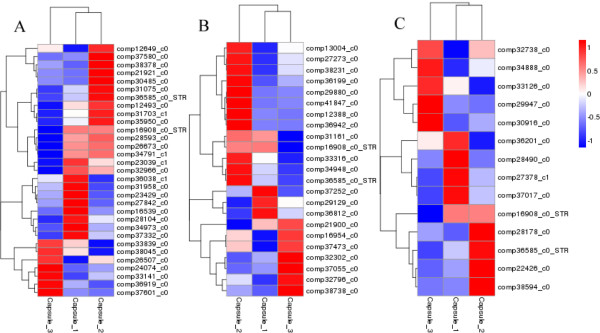
**Expression profiles clustering of CYP450, methyltransferase and isomerase genes from A to C respectively at three different capsule developmental stages.** Hierarchical clustering of expression data for 29 candidate CYP450 genes, 21 candidate methyltransferase genes and 12 candidate isomerase genes respectively using STR_KO_ as reference profiles. Expression ratios are expressed as Log 10 values.

STR is a starting point and key enzyme of TIAs synthesis involved in the early part of TIAs synthesis. *STR* expression will affect the metabolic activities of the downstream branch. The gene expression profiles of TIAs downstream branch are expected to be similar to the expression profiles of *STR*. The gene expression profiles not only include up/down-regulated expression, but also include gene expression variation and amplitude from each stage of capsule 1 → capsule 2 → capsule 3. Expression profiles of CYP450, methyltransferase and isomerase hierarchically clustered with that of STR (*STR*_Nr_, comp16908_c0 and comp38465_c1; *STR*_KO_, comp16908_c0 and comp36585_c0) (shown in Figures 8 and 9). Ten candidate genes were found to cluster closely with *STR* (correlation >0.99). The candidate genes of CYP450 had 4 unigenes (comp31075_c0, comp28593_c0, comp34791_c1 and comp26673_c0); candidate genes of methyltransferase had 3 unigenes (comp31161_c0, comp33316_c0 and comp34948_c0) and candidate genes of isomerase had 3 unigenes (comp22426_c0, comp38594_c0 and comp28178_c0). Through the alignment of transcriptome database, the candidate unigene sequences and amino acid sequences of “late step genes” of RIN and IRN biosynthesis were obtained and showed in Additional file 1: Table S9.

### Quantitative PCR analysis of candidate TIA biosynthesis genes

**Figure 10:**
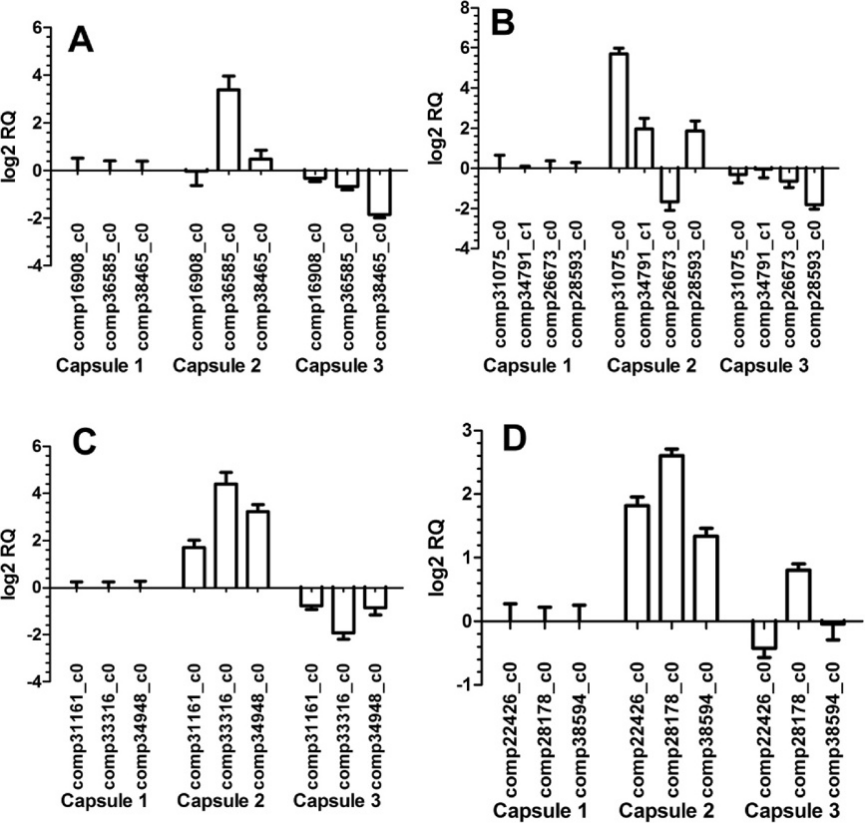
**Quantitative PCR analysis of the candidate downstream genes. A** STR; **B** CYP450; **C** Methyltransferases; **D** Isomerases.

Quantitative PCR analysis was performed on 13 selected genes from CYP450, methyltransferase, isomerases and STR functional categories putatively involved in TIA biosynthesis of *Uncaria*. The relative expression changes of the candidate genes are shown in Figure 10. Generally during the developmental process of capsule 1 to capsule 2, and capsule 2 to capsule 3 growth, the expression profiles of the genes assayed show highest gene expression at the capsule 2 stage, and lowest gene expression at capsule 3, mirroring the measured RIN and IRN content profiles, and confirming the DGE expression data. The STR genes (comp16908_c0, and comp38465_c1) however showed only marginal increases at capsule 2 stage. The relative expression of one of the CYP450 genes (comp26673_c0) was inverse to the expected expression, with lowest expression at capsule 2 and then increasing at capsule 3 (Figure 10 and Additional file 1: Table S8).

## Discussion

The medicinal parts of *Uncaria* are its “stem and stem hook” and the effective pharmacological constituents are RIN and IRN. In *Uncaria* plants, if a branch has a capsule, then there will be no stem hook. A branch with a stem hook growing on it, will not have flowers nor capsules next to it. In such case, there are two kinds of leaves and stems, one is the twig containing the capsule and the other is the twig containing a stem hook. “Stem and stem hook”, leaf, stem and capsule of *Uncaria* contain RIN and IRN, and different parts contain different content (data not shown). However, the difference between the RIN and IRN content of the “stem and stem hook” and that of stem on the branch with capsule is not significant. In addition, the serious lignification of the stem influences the integrity of RNA. By contrast, the difference between the RIN and IRN content of leaf on the branch with capsule and that of the leaf on the branch with stem hook is significant. No proteins related to the biosynthesis of the two TIAs have been found through dimensional electrophoresis analysis (data not shown). Among all the tissues and organs, the capsule has the highest RIN and IRN content and during the developmental process there is an increase and then decrease in the accumulation of the RIN and IRN. These changes in RIN and IRN content in the capsule reflect different rates of synthesis and provides a means for identifying candidate genes involved in the biosynthesis of RIN and IRN through analyzing the gene expression profiles [60].

Next generation sequencing technology (NGS) offers a shortcut for research on functional genomics, comparative genomics [61–63] and genetic analysis [64] of non-model plants. With the progress of NGS, and the increase of sequencing throughput, improvements in accuracy, and decreased costs, enable this technology to be broadly applied to various research areas [65, 66]. There is little to no genome information of *Uncaria* or the related species. Therefore sequencing RNA using NGS from a specific period of plant development or treatment provides the information necessary for de novo assembly of the transcriptome; generating a reference transcriptome sequence for non model plants that can provide the basis for finding genes associated with particular important functions [67–69]. DGE can quickly and thoroughly analyze the gene expression under a variety of tissues and conditions [70]. DGE however requires a reference sequence to align the relative small read lengths. Combining DGE with an RNA-sequencing generated reference transcriptome provides an excellent combination for analysis of non model plant species. Confirmation of the expression profiles of a subset of genes identified DGE using qPCR indicates the accuracy of this method for quickly identifying candidates based on expression profiles.

Predicting the biosynthesis pathway of RIN and IRN lays the theoretical foundation for the regulation of the RIN and IRN accumulation as well as their metabolic engineering research [71]. The formation of TIAs precursor involves the shikimate pathway of tryptophan metabolism and MEP pathway which are the common pathways of terpene biosynthesis. During the comparison and annotation of TIA like genes, we found all the encoding genes of the related enzymes in the two pathways in our cDNA library. Moreover, the encoding genes of STR which include the CYP450, methyltransferase and isomerase of later biosynthetic step in RIN and IRN biosynthesis were also found. By regulating the metabolic pathway, the accumulation of the RIN and IRN in the plant can be improved [72, 73]. This can be started from many aspects such as improving the TIAs biosynthesis pathway activity and upstream shunt volume of carbon skeleton. Improvement of biosynthesis pathway activity can be fulfilled through improving the activity of the key enzyme in the pathway [74, 75]. The increase of carbon shunt volume needs to shunt more carbon from the glycometabolism to enter the target pathway. Additionally, weaken or cutting off the carbon channel of non target pathways etc. The MEP pathway exists in the plastid, the amount of which can be increased by employing the matrilineal inheritance feature of plastid. In this way, more carbon in the glycometabolism will enter the RIN and IRN biosynthesis pathway. Alternatively, increase/enhance the transporter quantity/transport ability on the plastid membrane involved in the intermediate products of the MEP pathway of RIN and IRN biosynthesis through transformation of the organelle. Ultimately the substrate amount of iridoid pathway is increased. The auto-regulation mechanism of plant biosynthetic pathways is complex and strict. Construction of the vitro RIN and IRN biosynthesis pathway using information from the related research on vinblastine and vincristine [76–79] may provide a means of accelerating our knowledge of the pathways. The low yield of natural active substances is one of the factors limiting the development and utilization of Chinese medicine resources. The use of engineering techniques to improve production of natural active substance is an effective way to solve the contradiction between supply and demand. This study presented the pathway of RIN and IRN biosynthesis, and laid the theoretical foundation for the medicinal potential development of RIN and IRN. If the yield issues of RIN and IRN are effectively solved then they will have a wider range of applications in the treatment of Alzheimer’s disease.

## Conclusion

A combination of *De novo* transcriptome sequencing and DGE analysis based on the next generation sequencing technology was shown to be a powerful method for identifying candidate genes encoding enzymes responsible for the biosynthesis of novel secondary metabolites in a non-model plant. Through this method, biosynthetic encoding genes of *Uncaria* TIAs biological precursor were found. CYP450, methyltransferase and isomerase were selected as potential candidates involved in late biosynthesis of RIN and IRN. The transcriptome data from this study provides an important resource for understanding the formation of major bioactive constituents in the capsule extract from *Uncaria*.

## Methods

### Sample collection and preparation

*Uncaria* was collected from Nanning, Guangxi, China, and identified as *Uncaria rhynchophylla* by Ma Xiaojun. From blossom to ripened capsules, the capsules were collected once every ten days and six times totally. The collected capsules were divided into two parts. One part was frozen in liquid nitrogen for isolation of RNA, and stored at -80°C until use. The other part was used for the determination of RIN and IRN content by high performance liquid chromatography after drying and crushing. The plant samples were stored in Plant Physiology Laboratory of Northeast Agricultural University.

The analysis results from high performance liquid chromatography showed a single peak curve of RIN and IRN content changes (Figure 2A) [80]. Sampling point 3 had the highest RIN and IRN content and was designated capsule 2. Sampling point 1 (designated capsule 1) and 5 (designated capsule 3) had the low RIN and IRN content. RNA samples from sample point 1 (capsule 1), 3 (capsule 2) and 5 (capsule 3) were used for high-throughput sequencing.2 μL from each of three samples were mixed for transcriptome sequencing. 4 μL from each of three samples were used for expression profile sequencing. The RNA samples from sample point 1, 3 and 5 were named capsule 1, capsule 2 and capsule 3 in expression profile sequencing. Representative images of sample point 1, 3 and 5 are shown in Figure 2B,C,D.

### RNA isolation, quantification and qualification

Total RNA of capsule 1, capsule 2 and capsule 3 were isolated using the improved CTAB method [81]. RNA degradation and contamination was monitored on 1% agarose gels. RNA purity was checked using the NanoPhotometer®spectrophotometer (IMPLEN, CA, USA). RNA concentration was measured using Qubit®RNA Assay Kit in Qubit®2.0 Flurometer (Life Technologies, CA, USA). RNA integrity was assessed using the RNA Nano 6000 Assay Kit of the Bioanalyzer 2100 system (Agilent Technologies, CA, USA).

### Sample preparation for sequencing

A total amount of 3 μg RNA per sample were used as input material for the RNA sample preparations. All four samples had RNA integrity number values above 8.0. Sequencing libraries were generated using Illumina TruSeq™RNA Sample Preparation Kit (Illumia, San Diego, USA) following manufacturer’s recommendations and four index codes were added to attribute sequences to each sample. Briefly, mRNA was purified from total RNA using poly-T oligo-attached magnetic beads. Fragmentation was carried out using divalent cations under elevated temperature in Illumina proprietary fragmentation buffer. First strand cDNA was synthesized using random oligonucleotides and SuperScript II. Second strand cDNA synthesis was subsequently performed using DNA Polymerase I and RNase H. Remaining overhangs were converted into blunt ends via exonuclease/polymerase activities and enzymes were removed. After adenylation of 3’ ends of DNA fragments, Illumina PE adapter oligonucleotides were ligated to prepare for hybridization. In order to select cDNA fragments of preferentially 200 bp in length the library fragments were purified with AMPure XP system (Beckman Coulter, Beverly, USA). DNA fragments with ligated adaptor molecules on both ends were selectively enriched using Illumina PCR Primer Cocktail in a 10 cycle PCR reaction. Products were purified (AMPure XP system) and quantified using the Agilent high sensitivity DNA assay on the Agilent Bioanalyzer 2100 system.

### Clustering and sequencing

The clustering of the index-coded samples was performed on a cBot Cluster Generation System using TruSeq PE Cluster Kit v3-cBot-HS (Illumia) according to the manufacturer’s instructions. After cluster generation, the library preparations were sequenced on an Illumina Hiseq 2000 platform.

### Quality control

Raw data (raw reads) of fastq format were firstly processed through our self-written perl scripts. In this step, clean data (clean reads) were obtained by removing reads containing adapter, reads containing ploy-N and low quality reads from raw data. At the same time, Q20, Q30, GC-content and sequence duplication level of the clean data were calculated. All the downstream analyses were based on clean data with high quality.

### Transcriptome assembly

Pair-end sequencing was performed. The left files (read 1 file) from all libraries/samples were pooled into one big left.fq file, and right files (read 2 file) into one big right.fq file. Transcriptome assembly was accomplished based on the left.fq and right.fq using Trinity [41] with min_kmer_cov set to 2 and all other parameters set default.

### Gene functional annotation

Gene function was annotated based on the following databases: Nr (NCBI non-redundant protein sequences); Nt (NCBI non-redundant nucleotide sequences); Pfam (Protein family); KOG/COG (Clusters of Orthologous Groups of proteins); Swiss-Prot (A manually annotated and reviewed protein sequence database); KO (KEGG Ortholog database); GO (Gene Ontology).

### Reads mapping to the reference genome

Sequencing-received raw image data was transformed by base calling into sequence data. Prior to mapping reads to the reference database, we filtered all sequences to remove low quality sequences. A preprocessed database of nucleotide tag was created using our transcriptome reference database. The clean reads of the samples were mapped to the reference sequence of *Uncaria* transcriptome data, and obtained from the annotation information of each sample using RSEM software.

### Quantification of gene expression level

HTSeq v0.5.3 was used to count the reads numbers mapped to each gene. And then RPKM of each gene was calculated based on the length of the gene and reads count mapped to this gene. RPKM, Reads Per Kilobase of exon model per Million mapped reads, considers the effect of sequencing depth and gene length for the reads count at the same time, and is currently the most commonly used method for estimating gene expression levels [47].

### Differential expression analysis

Prior to differential gene expression analysis, for each sequenced library, the read counts were adjusted by edgeR program package through one scaling normalized factor. Differential expression analysis of two conditions was performed using the DEGseq R package (1.12.0). The P values were adjusted using the Benjamini & Hochberg method [51]. Corrected P-value of 0.005 and log2 (Fold change) of 1 were set as the threshold for significantly differential expression.

### GO and KEGG enrichment analysis of differentially expressed genes

Gene Ontology (GO) enrichment analysis of differentially expressed genes was implemented by the GOseq R package, in which gene length bias was corrected. GO terms with corrected P-value less than 0.05 were considered significantly enriched by differential expressed genes.

KEGG is a database resource for understanding high-level functions and utilities of the biological system, such as the cell, the organism and the ecosystem, from molecular-level information, especially large-scale molecular datasets generated by genome sequencing and other high-throughput experimental technologies (http://www.genome.jp/kegg/). We used KOBAS software to test the statistical enrichment of differential expression genes in KEGG pathways.

### Quantitative PCR

RNA for quantitative PCR analysis was the same samples as used for DGE. Primers were designed using Primer 5.0, and shown in Additional file 1: Table S10. Reverse transcription used PrimerScoript RT reagent Kit With gDNA eraser (perfect Real Time) (TaKaRa, RR047A) and Random 6 mers primer obtained cDNA. The standard curve was constructed using cDNA generated 1:5 with H_2_O. The cDNA was diluted in the 1:20 ratio as the sample reaction solution.

Quantitative PCR reactions used ABI7500 quantitative PCR, and the relative standard curve method was adopted to analyze the relative expression of genes. This study used SYBR® Premix Ex TaqTM II (TaKaRa, DRR820A) kit, which reaction system was 25 μL, final concentration of primers was 0.2 μmol · L^-1^, template was 1.0 μL, ROX Reference Dye II was 0.5 μL, H_2_O was used for complement system, and set up six repeats. Negative control used 1.0 μL water instead of the template. Reaction conditions were 95°C for 5 s in order to activate enzyme reaction. Two step cycles were then used; 95°C for 5 s, then 60°C for 30 s, 40 cycles; solubility curve conditions were 95°C for 15 s, 60°C for 1 min, 95°C for 30 s, 60°C for 15 s.

### Availability of supporting data

The data sets supporting the results of this article are available in the BioProject (BioProject:PRJNA222278, PRJNA222280, PRJNA222281, PRJNA222282) repository of the National Center for Biotechnology Information: http://www.ncbi.nlm.nih.gov/bioproject/?term=222278, http://www.ncbi.nlm.nih.gov/bioproject/?term=222280, http://www.ncbi.nlm.nih.gov/bioproject/?term=222281, http://www.ncbi.nlm.nih.gov/bioproject/?term=222282.

## Electronic supplementary material

Additional file 1: Table S1: Summary for the *Uncaria rhynchophylla* transcriptome. **Table S2.** The length statistics of the assembled transcripts and unigenes. **Table S3.** Coding genes and CDS involved in TIAs biosynthesis by Nr database annotation. **Table S4.** (A) Coding genes of cytochrome P450 protein. (B) Coding genes of methyltransferase. (C) Coding genes of isomerase. **Table S5.** Encoding genes involved in TIAs biosynthesis by KO database annotation. **Table S6.** RPKM and up/down-regulated expression of encoding genes involved in TIAs biosynthesis by Nr database annotation. **Table S7.** Up/down regulated expression of encoding genes involved in TIAs biosynthesis by KO database annotation. **Table S8.** (A) Differential expression analysis of cytochrome P450 genes. (B) Differential expression analysis of methyltransferase genes. (C) Differential expression analysis of isomerase genes. **Table S9.** Unigene and amino acid sequences of RIN and IRN biosynthetic encoding genes in the late step. **Table S10.** The quantitative PCR primers of candidate genes. (ZIP 122 KB)

Additional file 2: Figure S1: Error rate distribution of Capsule transcriptome sequencing. The x-axis indicates the nucleotide position of reads. The y-axis indicates the single-base error rate. 1–100 bp is a first terminal sequencing error rate distribution for the two-terminal sequencing reads, 100–200 bp is the other terminal sequencing error rate distribution. **Figure S2.** G/C content distribution. 0–100 bp is the GC content distribution of the first reads sequencing in paired-end sequencing, 100–200 bp is the GC content distribution of the other reads sequencing. **Figure S3.** (A) GO classification of differentially expressed genes in capsule 2 vs capsule 1. (B) GO classification of differentially expressed genes in capsule 3 vs capsule 1. (C) GO classification of differentially expressed genes in capsule 3 vs capsule 2. **Figure S4.** (A) KEGG pathway enrichment scatter diagram in capsule 2 vs capsule 1. (B) KEGG pathway enrichment scatter diagram in capsule 3 vs capsule 1. (C) KEGG pathway enrichment scatter diagram in capsule 3 vs capsule 2. (ZIP 2 MB)

## Abbreviations

RIN: Rhynchophylline
IRN: Isorhynchophylline
TIAs: Terpene indole alkaloids
Nr: Non-redundant protein sequences
KO: Kyoto Encyclopedia of Genes and Genomes Ortholog database
CYP450: Cytochrome P450
DGE: Digital gene expression profile
Aβ: β-amyloid protein
MEP/DOXP: Pathway triose phosphate/pyruvate or “non-mevalonate” pathway
IPP: Isopentenyl diphosphate
G10H: Geraniol-10-hydroxylase
LAMT: Loganic acid methyltransferase
SLS: Secologanin synthase
STR: Strictosidine synthase
Swiss Prot: A manually annotated and reviewed protein sequence database
Nt: NCBI nucleotide sequences
Pfam: Protein family
COG: Clusters of Orthologous Groups of proteins
GO: Gene Ontology
ORF: Open Reading Frame
CDS: Coding Sequence
TDC: Tryptophan decarboxylase
Ref: Reference sequence
RPKM: Reads Per Kilo bases per Million mapped Reads.
