# Supplementary material for: De novo transcriptome sequencing and digital gene expression analysis predict biosynthetic pathway of rhynchophylline and isorhynchophylline from Uncaria rhynchophylla, a non-model plant with potent anti-alzheimer’s properties
Source: BMC Genomics. 2014 Aug 12;15(1):676. doi: 10.1186/1471-2164-15-676 (PMC4143583; doi:10.1186/1471-2164-15-676)
Supplement: Supplementary file 1 — Additional file 1: Table S1: Summary for the Uncaria rhynchophylla transcriptome. Table S2. The length statistics of the assembled transcripts and unigenes. Table S3. Coding genes and CDS involved in TIAs biosynthesis by Nr database annotation. Table S4. (A) Coding genes of cytochrome P450 protein. (B) Coding genes of methyltransferase. (C) Coding genes of isomerase. Table S5. Encoding genes involved in TIAs biosynthesis by KO database annotation. Table S6. RPKM and up/down-regulated expression of encoding genes involved in TIAs biosynthesis by Nr database annotation. Table S7. Up/down regulated expression of encoding genes involved in TIAs biosynthesis by KO database annotation. Table S8. (A) Differential expression analysis of cytochrome P450 genes. (B) Differential expression analysis of methyltransferase genes. (C) Differential expression analysis of isomerase genes. Table S9. Unigene and amino acid sequences of RIN and IRN biosynthetic encoding genes in the late step. Table S10. The quantitative PCR primers of candidate genes. (ZIP 122 KB) [file 12864_2013_6370_MOESM1_ESM.zip › 3402726471070068_add10.doc]

Table S6 RPKM and up/down-regulated expression of encoding genes involved in TIAs biosynthesis by Nr database annotation

| Gene | Unigene ID | Capsule 1 | Capsule 2 | Capusle 3 | Capusle2  Vs  capusle1 | Capusle3  Vs  capusle2 | Capusle3  Vs  capusle1 |
| --- | --- | --- | --- | --- | --- | --- | --- |
| *AS* | comp12708_c0 | 60.47907439 | 125.0196131 | 25.48134953 | + | - | - |
| *TDC* | comp34949_c0 | 95.626756 | 79.15637915 | 15.93415708 |  | - | - |
| *DXS* | comp12371_c0  comp34786_c0 | 236.1717262  318.1370888 | 85.37772804  129.5445294 | 42.47306222  197.6461425 | -  - |  | - |
| *GPPS* | comp22220_c0  comp37903_c0 | 92.42386406  68.33661223 | 41.24245089  25.67543754 | 58.13358083  66.0502072 | -  - | + |  |
| *G10H* | comp33830_c0 | 18.67285547 | 70.73450815 | 25.87230894 | + | - |  |
| *LAMT* | comp33316_c0 | 19.50629182 | 124.2395761 | 2.42348996 | + | - | - |
| *SLS* | comp12327_c0  comp35216_c0  comp36839_c1  comp38387_c0 | 378.5539821  41.06731252  75.83118404  5.677360114 | 610.0621554  10.08302111  26.51939509  5.442189319 | 124.0852278  77.12069841  56.43251457  23.43303963 | -  - | -  +  +  + | +  + |
| *STR* | comp16908_c0  comp21960_c0  comp32940_c0  comp36585_c0  comp38465_c1 | 32.55753652  23.3381466  24.51728903  10.38594014  144.3959407 | 31.43533169  8.027305013  31.53672709  25.54539116  323.3885566 | 1.830674605  29.43799858  79.40323488  6.829921396  108.2380645 | + | -  +  +  -  - | - |

“+” indicates significantly up-regulated expression；“-” indicates significantly down-regulated expression.
