# Supplementary material for: De novo transcriptome sequencing and digital gene expression analysis predict biosynthetic pathway of rhynchophylline and isorhynchophylline from Uncaria rhynchophylla, a non-model plant with potent anti-alzheimer’s properties
Source: BMC Genomics. 2014 Aug 12;15(1):676. doi: 10.1186/1471-2164-15-676 (PMC4143583; doi:10.1186/1471-2164-15-676)
Supplement: Supplementary file 1 — Additional file 1: Table S1: Summary for the Uncaria rhynchophylla transcriptome. Table S2. The length statistics of the assembled transcripts and unigenes. Table S3. Coding genes and CDS involved in TIAs biosynthesis by Nr database annotation. Table S4. (A) Coding genes of cytochrome P450 protein. (B) Coding genes of methyltransferase. (C) Coding genes of isomerase. Table S5. Encoding genes involved in TIAs biosynthesis by KO database annotation. Table S6. RPKM and up/down-regulated expression of encoding genes involved in TIAs biosynthesis by Nr database annotation. Table S7. Up/down regulated expression of encoding genes involved in TIAs biosynthesis by KO database annotation. Table S8. (A) Differential expression analysis of cytochrome P450 genes. (B) Differential expression analysis of methyltransferase genes. (C) Differential expression analysis of isomerase genes. Table S9. Unigene and amino acid sequences of RIN and IRN biosynthetic encoding genes in the late step. Table S10. The quantitative PCR primers of candidate genes. (ZIP 122 KB) [file 12864_2013_6370_MOESM1_ESM.zip › 3402726471070068_add14.doc]

Table S10 The quantitative PCR primers of [candidate](javascript:void(0);) genes

| Gene name | Primer name | Primer sequence (5'to3') | Expected amplication  length (bp) |
| --- | --- | --- | --- |
| Reference gene | GADPH-1 | TCAAATCAACAACGGAGACG | 204 |
|  | GADPH-2 | GCCACGCAACTACTGCTACAC |  |
| Cytochrome P450 | comp31075-1 | CAAAACATCGTCCACCTTCCC | 292 |
|  | comp31075-2 | TGCCTCCAATAATCGCCGT |  |
|  | comp34791-1 | GAGCCAAAGCAGGAGGTGTC | 271 |
|  | comp34791-2 | CCTACTTATCGCAGAGCCATTCA |  |
|  | comp26673-1 | CACTCAAGTTGTAACGGAAGCC | 299 |
|  | comp26673-2 | TGATTAGGTGATGATGAACCAGAA |  |
|  | comp28593-1 | GGAAAGTTTGGTGAATGAAGGGTA | 215 |
|  | comp28593-2 | GGTCGTCATCAGCAGCAGGA |  |
| Methyltransferase | comp31161-1 | CCAGTGAAACGACCAATACCAG | 196 |
|  | comp31161-2 | CTAAAGGACAAGAGCGTGAGATG |  |
|  | comp33316-1 | GAATCACCATCAACCCTCCTC | 197 |
|  | comp33316-2 | TGCACTTCACTGGCTATCCAA |  |
|  | comp34948-1 | ATTAGTTGATGTTGGTGGTGGC | 268 |
|  | comp34948-2 | TTTTCCGTGGTCTGGTAGAGC |  |
| Isomerase | comp22426-1 | CGAAGGTAAAGGTGGAGAATGC | 258 |
|  | comp22426-2 | CCAAGTATGTAAGGCGAAGAGAT |  |
|  | comp28178-1 | TCCAAGTGGTGACTGGGTGAA | 225 |
|  | comp28178-2 | ATCGTTACAGGTCCCTTTGAGAA |  |
|  | comp38594-1 | ACAAAGGCTTCTTCGTGGCA | 247 |
|  | comp38594-2 | GAAACAAATAATGTAGTTGAGGTGGC |  |
| STR | comp16908-1 | GGGAGGGTCTACGACTTGGG | 204 |
|  | comp16908-2 | GCTGCTAACTTCCGTGAAATAAACA |  |
|  | comp36585-1 | GCGACGGCAACAGGGA | 224 |
|  | comp36585-2 | GCCAGTAACAGGGTCAACG |  |
|  | comp384651-1 | CAACTTCCGACGAATAAAATGCTT | 154 |
|  | comp384651-2 | TGCCGAATGGGAGTAGTAATCAA |  |
