# Supplementary material for: De novo transcriptome sequencing and digital gene expression analysis predict biosynthetic pathway of rhynchophylline and isorhynchophylline from Uncaria rhynchophylla, a non-model plant with potent anti-alzheimer’s properties
Source: BMC Genomics. 2014 Aug 12;15(1):676. doi: 10.1186/1471-2164-15-676 (PMC4143583; doi:10.1186/1471-2164-15-676)
Supplement: Supplementary file 1 — Additional file 1: Table S1: Summary for the Uncaria rhynchophylla transcriptome. Table S2. The length statistics of the assembled transcripts and unigenes. Table S3. Coding genes and CDS involved in TIAs biosynthesis by Nr database annotation. Table S4. (A) Coding genes of cytochrome P450 protein. (B) Coding genes of methyltransferase. (C) Coding genes of isomerase. Table S5. Encoding genes involved in TIAs biosynthesis by KO database annotation. Table S6. RPKM and up/down-regulated expression of encoding genes involved in TIAs biosynthesis by Nr database annotation. Table S7. Up/down regulated expression of encoding genes involved in TIAs biosynthesis by KO database annotation. Table S8. (A) Differential expression analysis of cytochrome P450 genes. (B) Differential expression analysis of methyltransferase genes. (C) Differential expression analysis of isomerase genes. Table S9. Unigene and amino acid sequences of RIN and IRN biosynthetic encoding genes in the late step. Table S10. The quantitative PCR primers of candidate genes. (ZIP 122 KB) [file 12864_2013_6370_MOESM1_ESM.zip › 3402726471070068_add7.doc]

Table S3 Coding genes and CDS involved in TIAs biosynthesis by Nr database annotation

| Gene | Unigene ID | Query length | NR evalue | len | frame | CDS start | CDS end |
| --- | --- | --- | --- | --- | --- | --- | --- |
| AS | comp12708_c0  comp16364_c0  comp22155_c0  comp22155_c1  comp35643_c0  comp63983_c0  comp125706_c0 | 2288  1798  283  289  721  466  410 | 0  0  3.49E-31  9.34E-58  1.73E-50  1.25E-47  1.26E-57 | 1863  1287  210  288  345  351  408 | 1  3  2  -2  1  -1  2 | 91  147  74  288  1  466  2 | 1953  1433  283  1  345  116  409 |
| TDC | comp34200_c0  comp34200_c1  comp34949_c0 | 918  791  1089 | 2.32E-134  9.63E-73  0 | 783  429  1089 | 1  1  1 | 136  1  1 | 918  429  1089 |
| DXS | comp12371_c0  comp33166_c0  comp33166_c1  comp33166_c2  comp34786_c0  comp36275_c0  comp106790_c0  comp110780_c0  comp131107_c0  comp216472_c0 | 849  544  1034  1647  1182  2448  441  239  242  231 | 7.41E-57  1.01E-39  0  0  0  0  1.74E-91  2.77E-40  7.53E-46  2.94E-27 | 411  234  837  1359  1068  2148  438  237  240  231 | -2  -2  -2  3  -2  2  2  2  -1  1 | 848  543  1033  288  1070  95  2  2  242  1 | 438  310  197  1646  3  2242  439  238  3  231 |
| DXR | comp25807_c0 | 1959 | 0 | 1440 | 3 | 177 | 1616 |
| CMS | comp35762_c1 | 941 | 5.35E-30 | 192 | -2 | 502 | 311 |
| CMK | comp16877_c0 | 1765 | 0 | 1323 | 2 | 143 | 1465 |
| HDS | comp16899_c1 | 233 | 1.25E-43 | 231 | 2 | 2 | 232 |
| GPPS | comp22220_c0  comp24021_c0  comp29372_c0  comp37903_c0  comp79687_c0 | 1688  476  1559  1275  261 | 0  1.18E-45  2.98E-178  3.67E-82  9.26E-15 | 1233  474  1221  915  114 | -1  2  -1  3  3 | 1505  2  1490  63  3 | 273  475  270  977  116 |
| G10H | comp22971_c0  comp33830_c0  comp34840_c0  comp78202_c0 | 214  1722  1443  383 | 2.72E-11  0  0  1.02E-40 | 213  1512  1200  96 | -2  -1  -1  1 | 213  1722  1443  286 | 1  211  244  381 |
| LAMT | comp12702_c0  comp24149_c0  comp26296_c0  comp26296_c1  comp33316_c0  comp46474_c0 | 351  796  241  241  1555  215 | 5.73E-08  1.61E-16  3.65E-41  3.50E-26  0  3.75E-32 | 99  204  240  240  1146  213 | 2  2  1  2  -2  -3 | 2  2  1  2  1494  213 | 100  205  240  241  349  1 |
| SLS | comp12327_c0  comp21220_c0  comp23918_c0  comp26207_c0  comp27706_c0  comp28672_c2  comp30318_c0  comp30911_c0  comp35216_c0  comp36839_c1  comp38387_c0  comp38415_c0  comp165797_c0 | 2048  211  908  419  887  366  1846  343  1876  1925  1824  1963  217 | 0  2.42E-34  7.47E-114  3.17E-57  1.71E-42  2.79E-28  0  2.04E-10  0  0  1.90E-178  0  2.67E-18 | 1587  210  906  417  138  180  1554  240  1605  1602  432  1566  216 | -3  1  3  2  -2  2  -2  2  1  -1  1  -1  2 | 1872  1  3  2  412  2  1713  2  10  1877  1  1798  2 | 286  210  908  418  275  181  160  241  1614  276  432  233  217 |
| STR | comp7830_c0  comp16908_c0  comp20385_c0  comp21960_c0  comp28394_c0  comp32940_c0  comp34778_c0  comp34785_c0  comp36585_c0  comp38465_c0  comp38465_c1  comp50084_c0  comp69380_c0  comp122990_c0  comp194171_c0  comp201355_c0  comp303655_c0 | 343  1332  292  1117  1174  1315  1821  1472  1255  409  657  1202  714  325  265  202  209 | 1.89E-16  3.22E-138  3.50E-35  8.03E-86  1.74E-99  1.07E-132  0  0  1.38E-89  4.15E-08  7.92E-49  1.79E-126  7.81E-37  6.76E-36  2.24E-27  7.79E-13  2.25E-13 | 339  1062  291  1035  1032  1221  1179  1224  1155  96  336  1041  540  321  198  201  147 | 3  1  -1  -2  Minus  -2  -3  1  3  2  3  -1  -2  -3  -2  2  -3 | 3  46  292  1116  1174  1314  1561  1  3  2  3  1202  713  323  264  2  207 | 341  1107  2  82  143  94  383  1224  1157  97  338  162  174  3  67  202  61 |
