# Supplementary material for: De novo transcriptome sequencing and digital gene expression analysis predict biosynthetic pathway of rhynchophylline and isorhynchophylline from Uncaria rhynchophylla, a non-model plant with potent anti-alzheimer’s properties
Source: BMC Genomics. 2014 Aug 12;15(1):676. doi: 10.1186/1471-2164-15-676 (PMC4143583; doi:10.1186/1471-2164-15-676)
Supplement: Supplementary file 1 — Additional file 1: Table S1: Summary for the Uncaria rhynchophylla transcriptome. Table S2. The length statistics of the assembled transcripts and unigenes. Table S3. Coding genes and CDS involved in TIAs biosynthesis by Nr database annotation. Table S4. (A) Coding genes of cytochrome P450 protein. (B) Coding genes of methyltransferase. (C) Coding genes of isomerase. Table S5. Encoding genes involved in TIAs biosynthesis by KO database annotation. Table S6. RPKM and up/down-regulated expression of encoding genes involved in TIAs biosynthesis by Nr database annotation. Table S7. Up/down regulated expression of encoding genes involved in TIAs biosynthesis by KO database annotation. Table S8. (A) Differential expression analysis of cytochrome P450 genes. (B) Differential expression analysis of methyltransferase genes. (C) Differential expression analysis of isomerase genes. Table S9. Unigene and amino acid sequences of RIN and IRN biosynthetic encoding genes in the late step. Table S10. The quantitative PCR primers of candidate genes. (ZIP 122 KB) [file 12864_2013_6370_MOESM1_ESM.zip › 3402726471070068_add9.doc]

Table S5 Encoding genes involved in TIAs biosynthesis by KO database annotation

| ID | KO Description | EC | Number of unigene | Unigene ID |
| --- | --- | --- | --- | --- |
| K01662 | 1-deoxy-D-xylulose-5-phosphate synthase | 2.2.1.7 | 12 | comp12371_c0  comp33166_c0  comp33166_c1  comp33166_c2  comp34786_c0  comp36275_c0  comp87798_c0  comp106790_c0  comp110780_c0  comp111707_c0  comp131107_c0  comp216472_c0 |
| K00099 | 1-deoxy-D-xylulose-5-phosphate reductoisomerase | 1.1.1.267 | 1 | comp25807_c0 |
| K00991 | 2-C-methyl-D-erythritol 4-phosphate cytidylyltransferase | 2.7.7.60 | 1 | comp35762_c1 |
| K00919 | 4-diphosphocytidyl-2-C-methyl-D-erythritol kinase | 2.7.1.148 | 1 | comp16877_c0 |
| K01770 | 2-C-methyl-D-erythritol 2,4-cyclodiphosphate synthase | 4.6.1.12 | 1 | comp16605_c0 |
| K03526 | (E)-4-hydroxy-3-methylbut-2-enyl-diphosphate synthase | 1.17.7.1 | 3 | comp16899_c1  comp38313_c0  comp40533_c0 |
| K03527 | 4-hydroxy-3-methylbut-2-enyl diphosphate reductase | 1.17.1.2 | 2 | comp27824_c0  comp38982_c0 |
| K01823 | isopentenyl-diphosphate delta-isomerase | 5.3.3.2 | 3 | comp18737_c0  comp27378_c1  comp41463_c0 |
|  |  |  |  |  |
| K12467 | myrcene/ocimene synthase | 4.2.3.15 | 1 | comp3443_c0 |
|  |  |  |  |  |
| K01626 | 3-deoxy-7-phosphoheptulonate synthase | 2.5.1.54 | 6 | comp16248_c0  comp16248_c1  comp16624_c0  comp21495_c0  comp30078_c0  comp36517_c0 |
| K01735 | 3-dehydroquinate synthase | 4.2.3.4 | 2 | comp25052_c0  comp35080_c0 |
| K13832 | 3-dehydroquinate dehydratase /  shikimate dehydrogenase | 4.2.1.10 1.1.1.25 | 6 | comp20762_c0  comp20762_c2  comp22817_c0  comp30703_c0  comp34602_c0  comp37202_c0 |
| K00891 | shikimate kinase | 2.7.1.71 | 2 | comp34045_c0  comp38048_c0 |
| K00800 | 3-phosphoshikimate 1-carboxyvinyltransferase | 2.5.1.19 | 1 | comp36951_c0 |
| K01736 | chorismate synthase | 4.2.3.5 | 2 | comp36944_c0  comp256004_c0 |
| K01657 | anthranilate synthase component I | 4.1.3.27 | 6 | comp12708_c0  comp22155_c1  comp35643_c0  comp63983_c0  comp125706_c0  comp16644_c0 |
| K00766 | anthranilate phosphoribosyltransferase | 2.4.2.18 | 1 | comp35688_c0 |
| K01817 | phosphoribosylanthranilate isomerase | 5.3.1.24 | 2 | comp32187_c0  comp43116_c0 |
| K01609 | indole-3-glycerol phosphate synthase | 4.1.1.48 | 1 | comp32640_c0 |
| K01695 | tryptophan synthase alpha chain | 4.2.1.20 | 4 | comp16565_c0  comp30047_c0  comp34549_c0  comp24923_c0 |
|  |  |  |  |  |
| K01757 | strictosidine synthase | 4.3.3.2 | 6 | comp16908_c0  comp28394_c0  comp36585_c0  comp69380_c0  comp122990_c0  comp201355_c0 |
