# Supplementary material for: De novo transcriptome sequencing and digital gene expression analysis predict biosynthetic pathway of rhynchophylline and isorhynchophylline from Uncaria rhynchophylla, a non-model plant with potent anti-alzheimer’s properties
Source: BMC Genomics. 2014 Aug 12;15(1):676. doi: 10.1186/1471-2164-15-676 (PMC4143583; doi:10.1186/1471-2164-15-676)
Supplement: Supplementary file 2 — Additional file 2: Figure S1: Error rate distribution of Capsule transcriptome sequencing. The x-axis indicates the nucleotide position of reads. The y-axis indicates the single-base error rate. 1–100 bp is a first terminal sequencing error rate distribution for the two-terminal sequencing reads, 100–200 bp is the other terminal sequencing error rate distribution. Figure S2. G/C content distribution. 0–100 bp is the GC content distribution of the first reads sequencing in paired-end sequencing, 100–200 bp is the GC content distribution of the other reads sequencing. Figure S3. (A) GO classification of differentially expressed genes in capsule 2 vs capsule 1. (B) GO classification of differentially expressed genes in capsule 3 vs capsule 1. (C) GO classification of differentially expressed genes in capsule 3 vs capsule 2. Figure S4. (A) KEGG pathway enrichment scatter diagram in capsule 2 vs capsule 1. (B) KEGG pathway enrichment scatter diagram in capsule 3 vs capsule 1. (C) KEGG pathway enrichment scatter diagram in capsule 3 vs capsule 2. (ZIP 2 MB) [file 12864_2013_6370_MOESM2_ESM.zip › 3402726471070068_add2.doc]

Figure S2 G/C content distribution.

0-100 bp is the GC content distribution of the first reads sequencing in paired-end sequencing, 100-200bp is the GC content distribution of the other reads sequencing.
